# Medical Selections from Herndon’s Report on the Valley of the Amazon

**Published:** 1854-05

**Authors:** 


### *Medical Selections from Herndon's Report on the Valley of the Amazon.*

Continuing our review of Lieut. Herndon's excellent work on the physical condition and resources of the Valley of the Amazon, we proceed to notice such other items in the course of the work, as may be of interest to the medical reader.

He gives numerous instances of the early maturity of the women in that region:

"The only inhabitants of the rancho seemed to be two little girls; but I found in the morning that one of them had an infant, though she did not appear to be more than twelve or thirteen years of age."

A novel mode of fishing is alluded to:

"The fishing-party of the padre was a large affair. They had four or five canoes, and a large quantity of barbasco. The manner of fishing is to close up the mouth of a inlet of the river with a net-work made of reeds, and then, mashing the barbasco root to a pulp, throw it into the water. This turns the water white, and poisons it, so that the fish soon commence rising to the surface dead, and are taken into the canoes with small tridents. Almost at the moment of throwing the barbasco into the water, the smaller fish rise to the surface and die in two or three minutes; the larger fish survive longer; and, therefore, a successful fishing of this sort is a matter of half a day, or till the canoes are filled."

He speaks of a vegetable growth that may yet be of eminent service, under enlightened hands, in the treatment of disease:

"I obtained at this place the sap of a large tree called *catao*, which is said to be very poisonous. It appears to be acrid, and acts like a powerful caustic. The man who chopped the bark, to let the sap run, always turned away his face as he struck, for fear of its getting into his eyes. The Indians employ it for the purpose of curing old dull sores. The tree is generally very large; has a smooth bark, but with knots on it bearing short thorns. The leaf is nearly circular; it is called in Brazil *assacu*, and is there thought to be a remedy for leprosy. We gathered also some leaves and root of a running plant called *guaco*, which, steeped in spirits, and applied internally and externally, is said to be an antidote to the bite of a snake. I think it probable that this may be a fancy of the Indians, originating from the fact that the leaf has something the appearance and color of a snake-skin. There is a great abundance of it all over the Montana."

But the resources of that region are dietetic as well as medicinal.

"Farinha is a general substitute for bread in all the course of the Amazon below the Brazilian frontier. It is used by all classes, and in immense quantities by the Indians and laborers. Our boatmen in Brazil were always contented with plenty of salt fish and farinha.— Every two or three hours of the day, whilst traveling, they would stop rowing, pour a little water upon a large gourd-full of farinha, and pass around the mass (which they called *pirao*) as if it were a delicacy.

The women generally make the farinha. They soak the root of the mandioc (Iatropha Manihot) in water till it is softened a little, when they scrape off the skin, and grate it upon a board smeared with some of the adhesive gums of the forest, and sprinkled with pebbles. The white grated mass is put into a conical-shaped bag, made of the course fibres of a palm, and called *tapiti*. The bag is hung up to a peg driven into a tree, or a post of the shed; a lever is put through a loop at the bottom of the bag; the short end of the lever is placed under a chock nailed to the post below, and the woman hangs her weight on the long end. This elongates the bag, and brings a heavy pressure upon the mass within, causing all the juice to ooze out through the interstices of the wicker-work of the bag.—

When sufficiently pressed the mass is put on the floor of a mud oven; heat is applied, and it is stirred with a stick till it granulates in very irregular grains, (the largest about the size of our No. 2 shot,) and is sufficiently toasted to drive off all the poisonous qualities which it has in a crude state. It is then packed in baskets (lined and covered with palm-leaves) of about sixty-four pounds weight, which are generally sold, all along the river, at from seventy-five cents to one dollar. The sediment of the juice which runs from the tapiti is tapioca, and is used to make custards, puddings, starch, &c."

Here followeth an old friend of ours, though more a friend of old Dr. Townsend *et al.*

"Sarsaparilla is a vine of sufficient size to shoot up fifteen or twenty feet from the root without support. It then embraces the surrounding trees, and spreads to a great distance. The main root sends out many tendrils, generally about two lines in diameter, and five feet long. These are gathered and tied up in large bundles of about a Portuguese arroba, or thirty-two pounds of weight. The main root, or *madre*, should not be disturbed; but the Indians are little careful in this matter, and frequently cut it off, by which much sarsaparilla is destroyed. The digging up of the small roots out of the wet and mashy soil is a laborious and unhealthy occupation.

It is to be found on the banks of almost every tributary of the great streams of the Montana; but a great many of these are not worked, on account of the savages living on their banks, who frequently attack the parties that come to gather it. The whole southern border of the Amazon, from the mouth of the Ucayali to that of the *Yavari*, is inhabited by savages, all averse to intercourse with the white man. The same is the case on the "Tigreyacu," where there is said to be much sarsaparilla. Padre Calvo, the president of the Missions at Sarayacu, told me that, although he has the exclusive right, by order of the prefect, of collecting all the sarsaparilla on the Ucayali and its tributaries, he could not, if I were willing to pay any price, supply me with more than three hundred arrobas per annum, on account of the difficulty of getting laborers who are willing to brave the attacks of the savages."

The sympathising Lieutenant tried his hand at practice, in an emergency, and succeeded precisely the same as some who try their hands at it, not in an emergency.

"The town is situated on a hill, with the forest well cleared away from around it, and is a healthy place. I saw only two cases of sickness during my stay of two weeks. They were acute cases of disease, to which people are liable everywhere. Both patients died; probably for want of medical attention. I gave the man who had the dysentery some doses of calomel and opium, (a prescription I had from Dr. Smith, of Lima;) but he died with the last dose. Though solicited, I would have nothing to do with the other case. It was a woman; and I had no confidence in my practice. I could only add my mite to a subscription raised by the whites for the benefit of her orphan children."

He quotes a good custom, but does not say that he does so as a mere hint to more civilized communities.

"These Indians are jealous, and punish conjugal infidelity with severity, and also departure from the laws of chastity on the part of the unmarried female."

We select an example of an unfortunate state of things, followed by a narration of a very convenient method of getting over a difficulty."

"I saw to-day twenty lazy rascals loitering about, whilst the same number of women were fetching earth and water, trampling it into mud, and plastering the walls of the convento with it. I also saw the women cleaning up and carrying away the weeds and bushes of the town; most of them, too, with infants hanging to their backs.—These marry very young. I saw some, whom I took to be children, with babies that I was told were their own. They suffer very little in parturition, and, in a few hours after the birth of a child, they bathe, go to the chacra, and fetch home a load of yuccas.

Most of them are able to perform all the offices of a midwife for themselves. I am told that sometimes, when a man and his wife are traveling together in a canoe, the woman will signify to her husband her desire to land; will retreat into the woods, and in a very short time return with a newly-born infant, which she will wash in the river, sling to her back, and resume her paddle again. Even the ladies of this country are confined a very short time. The mother of my little namesake was about her household avocations in seven days after his birth. This probably arises from three causes: the climate, the habit of wearing loose dresses, and the absence of dissipation."

